# Traditional Korean East Asian Medicines and Herbal Formulations for Cognitive Impairment

**DOI:** 10.3390/molecules181214670

**Published:** 2013-11-26

**Authors:** Hemant Kumar, Soo-Yeol Song, Sandeep Vasant More, Seong-Mook Kang, Byung-Wook Kim, In-Su Kim, Dong-Kug Choi

**Affiliations:** Department of Biotechnology, College of Biomedical and Health Science, Konkuk University, Chung-ju 380-701, Korea; E-Mails: hemantbhave@gmail.com (H.K.); go_world87@nate.com (S.-Y.S.); sandeepbcp@gmail.com (S.V.M.); big-_-bear@hanmail.net (S.-M.K.); kb62@lycos.co.kr (B.-W.K.); kis5497@kku.ac.kr (I.-S.K.)

**Keywords:** Traditional Korean Medicine, memory, herbal formulations, *Acorus gramineus*, Sasang Constitutional Medicine

## Abstract

*Hanbang*, the Traditional Korean Medicine (TKM), is an inseparable component of Korean culture both within the country, and further afield. Korean traditional herbs have been used medicinally to treat sickness and injury for thousands of years. Oriental medicine reflects our ancestor’s wisdom and experience, and as the elderly population in Korea is rapidly increasing, so is the importance of their health problems. The proportion of the population who are over 65 years of age is expected to increase to 24.3% by 2031. Cognitive impairment is common with increasing age, and efforts are made to retain and restore the cognition ability of the elderly. Herbal materials have been considered for this purpose because of their low adverse effects and their cognitive-enhancing or anti-dementia activities. Herbal materials are reported to contain several active compounds that have effects on cognitive function. Here, we enumerate evidence linking TKMs which have shown benefits in memory improvements. Moreover, we have also listed Korean herbal formulations which have been the subject of scientific reports relating to memory improvement.

## 1. Introduction

“Evidence-based medicine is the conscientious, explicit, and judicious use of current best evidence in making decisions about the care of individual patients” [1]. Traditional medicines (TMs) were developed with knowledge systems of different societies before the era of modern medicine. They were based on direct outcome/benefits in human, and differ from modern medicine which goes through the extensive process of drug discovery. Complementary and alternative medicine (CAM) has grown in worldwide popularity in the past 10 years [2,3]. CAM/TM/herbal medicines are preferred because they are believed to be safe and effective and many have cognitive-enhancing or anti-dementia activities [4,5].

The elderly population in Korea is increasing rapidly; the proportion of the population who are 65 or older accounted for 5.45 million persons in 2010, will increase 2.3-fold to 12.69 million persons in 2030, and 3-fold to 17.62 million persons in 2060 [6]. With increasing investment in healthcare, the average life span is also anticipated to rise. Male’s life expectancy at birth would increase from 77.2 years in 2010 to 86.6 years in 2060. For females, life expectancy at birth would rise from 84.1 years in 2010 to 90.3 years in 2060 [6]. With such a trend, there is an increasing emphasis on how to live a healthy life, rather than merely sustaining life.

Cognitive impairment is one of the most imperative features of geriatric conditions, and presents a major health problem in an aging society. Successful treatments for dementia have been developed from herbal drugs in the past. Galantamine (an alkaloid obtained from the bulbs and flowers of the Caucasian snowdrop *Galanthus woronowii*) [7,8] and huperzine A (a sesquiterpene alkaloid found in the firmoss *Huperzia serrata*) [9,10] are some of the standard drugs which exhibit benefits in cognitive impairment. The use of herbal remedies, particularly ginseng, has become increasingly popular for improving cognitive performance in recent years. In addition, herbal materials are reported to contain several active compounds which have effects on cognitive function, resulting from the fact that cognitive dysfunctions are not solely dependent on the decreased activity of cholinergic neurotransmitter systems [11]. Here, we enumerate evidence linking traditional Korean plants which have shown benefits in memory improvement. Moreover, we have also listed Korean herbal formulations which have been the subject of scientific reports relating to memory improvement.

## 2. History and Present Status of TKM

*Hanbang*, the traditional Korean medicine (TKM), is an inseparable component of Korean culture both within Korea, and throughout Korean communities worldwide [12]. Koreans have traditionally used herbs for the treatment of sickness and injury. The period of Joseon (1392–1910) brought the first encyclopedia of oriental medicine [13] which summarized Korean oriental medicine, and later, Sasang Constitutional Medicine (SCM) was established under the principles of Korean medical tradition [14]. It categorizes humans into four unique constitution types: Taeyangyin (TY Type), Tae-eumin (TE Type), Soyangyin (SY Type) and Soeumin (SE Type) depending on the nature of an individual’s physiological, psychological and physical characteristics, that lead to differential responses to herbs [14]. This classification is based on assumptions that invariant constitution is determined at birth and some remedies are only appropriate for certain constitutions and can cause adverse effects in others. This phenotypic variation predicts an underlying genetic basis for SCM [15]. Cumulative scientific evidence proposes a genetic basis for SCM. For these reasons, it can be concluded that oriental medicine is a traditional science in Korea, which reflects our ancestor’s wisdom and experience, and which can be correlated with today’s scientific evidence.

Korea has highest percentage (15.26%) of TM doctors in hospitals and clinics in East Asia, followed by mainland China (12.63%), and the Taiwan region (9.69%) [16]. Several factors are responsible for placing Oriental medicine in competition with Western medicine in Korea. One reason is that in Korea, Oriental medicine is legally institutionalized and its medical services are covered by National Health Insurance. Since 1987 acupuncture, moxibustion, and cupping are usually covered by medical insurance in Korea and most herbal extracts are covered, with the exception of decoctions of raw herbs [16]. Furthermore, Korea is a member of the WHO Pharmacovigilance Program for monitoring adverse drug reactions, including those related to the use of herbal drugs [16].

## 3. Cognitive Impairment in the Korean Elderly

The elderly, defined as being 65 years of age or older, made up just 3.8% of the Korean national population in 1980, but this proportion is expected to grow in coming years. The prevalence of overall dementia in Korea ranged from 6.3% to 13.0%, while estimates of the prevalence of Alzheimer’s disease (AD) ranged from 4.2% to 9.0%, and those of vascular dementia (VD) ranged from 1.0% to 4.8%. Almost all studies carried out in Korea have reported an increased risk of AD with advanced age [17].

There is growing evidence that subtle losses in cognitive function may be characteristic of a transition to early AD. Current research is directed on the identification of those individuals with mild cognitive impairment (MCI) who are most likely to translate to AD. MCI encompasses more significant cognitive and memory decline than normal aging, and exemplifies a significant risk factor for the development of dementia [18]. In one study, the prevalence of dementia, MCI and the factors associated with risk of dementia among a representative nationwide sample of Korean elderly people (65 years or older) were investigated. AD was the most prevalent type (5.7%), followed by VD (2.0%). The amnestic subtype (20.1%) was much more common than the non-amnestic subtype of MCI (4.0%). It was reported that number of dementia patients is expected to double every 20 years until 2050 in Korea, and that AD is expected to account for progressively more dementia cases in the future [19]. In another study, the prevalence of dementia and its major subtypes in an elderly urban Korean population were estimated. The estimated age and gender-standardized occurrences were 6.3% for dementia, 4.8% for AD, 1.0% for VD, and 0.4% for dementia with Lewy bodies (DLB). The prevalence of AD increased consistently with age, whereas that of VD peaked at age 75–79 years, and decreased thereafter. Of the dementia patients, 72.0% were classified as having very mild or mild stages of the disease. The prevalence of dementia in a typical urban area of Korea was estimated to be 6.3%, and AD was the most prevalent subtype. DLB is less prevalent than VD among these community-dwelling Korean elders [20].

## 4. Evidence-Linked Herbal Plants of TKM for Memory Improvement

In Korean, “geonmang” is the term referred as forgetfulness, amnesia or poor memory. Several plants like *Panax ginseng*, *Poria cocos*, and *Polygala tenuifolia* are described for the treatment of geonmang in the book Dong-eui-bo-gam.

### 4.1. Panax Ginseng

“Ginseng” (Fam. Araliaceae) is the dried root of several species of the plant genus *Panax*. It has a long medicinal history going back more than 5,000 years [21]. Ginseng is typically found in cooler climates such as Northern Hemisphere, North America and in Eastern Asia. The most widely used family member is *Panax ginseng*, which is indigenous to the Far East (most notably Korea). More than 200 ginsenosides and non-saponin constituents have, thus far, been isolated from and identified in ginseng. Among the numerous biological activities shown by ginseng, anti-aging, particularly nootropic effects are evident [22]. Among the 30 major ginsenosides, Rb1 and Rg1 are considered the main active ingredients of *Panax* species [23]. Rg1 improves learning and memory in normal rats and mice. The nootropic signaling pathway has also been assessed in normal rats, and the Rg1-induced signaling pathway is similar to memory formation that occurs in mammals, suggesting that Rg1 may potentially increase intellectual capacity in normal people [24]. Ginsenoside Rh2 recovers the scopolamine-induced reduction in long-term potentiation (LTP) in the hippocampal CA1 area [25]. Rb1 enhances the stimulatory effect of neurite outgrowth [26], promotes neurotransmitter release by modulating phosphorylation of synapsins through a cAMP-dependent protein kinase pathway [27], upregulates cell genesis in the dentate gyrus (DG) and CA3 hippocampal subregions [28]. Traditionally, two types of ginseng have been used: white ginseng, which is air-dried ginseng, and red ginseng, which is produced by steaming raw ginseng at 98–100 °C for 2–3 h [29]. Heat processes have been adopted for enhancing the pharmacological activities of ginseng, and these processes alter its chemical composition [30]. Red ginseng contains ginsenoside Rg5 as a main constituent [30,31]. Related to the memory deficit-protecting effects of ginsenosides, ginsenoside Rg3 and ginsenoside Rg5/Rk1 mixture (1:1, w/w), isolated from heat-processed ginseng, show a memory enhancing effect in scopolamine-treated mice [32]. Ginsenoside Rg3 isolated from red ginseng and its metabolite ginsenoside Rh2, as well as ginsenoside Rg1, also protected against scopolamine-induced memory deficit in mice [25,33]. However, when ginsenoside Rg5 was incubated with human feces, it was metabolized to ginsenoside Rh3 [32]. Recently, ginsenoside Rg5 and its metabolite ginsenoside Rh3, isolated from heated-processed ginseng treated with and without human feces, each showed memory improvement in mice by inhibiting acetylcholinesterase (AChE) activity, and increasing brain derived neurotrophic factor (BDNF) expression and cAMP response element-binding protein (CREB) activation [34]. A new type of processed ginseng named Sun ginseng (SG), containing higher levels of ginsenosides such as Rg3, Rg5 and Rk1 [30], has memory-enhancing activities and these effects are mediated, in part, by increasing cell survival and proliferation, as well as by increasing the levels of pERK and pAkt [35]. Moreover, gintonin, a novel lysophosphatidic acid (LPA)-ginseng protein complex has been isolated from ginseng. Gintonin activates G protein-coupled LPA receptors with high affinity. Gintonin is reported to mediate NMDA potentiation and LTP induction in the hippocampus, via the activation of LPA receptor. This effect might be responsible for learning and memory related brain functions of ginseng [36]. In a recent study, the effect of fermented ginseng (FG) on memory impairment and β-amyloid (Aβ) reduction was observed in *in vitro* and *in vivo* models of AD. FG extract treatment resulted in a significant recovery of memory function in a scopolamine-injected ICR mouse model, and in a transgenic mouse model. Brain soluble Aβ levels, measured from the cerebral cortex of transgenic mice, were significantly reduced by FG extract treatment [37]. Several studies have indicated that the administration of ginseng may improve learning and memory in healthy volunteers [38,39].

### 4.2. Polygala Tenuifolia

*Polygala tenuifolia* (Fam. Polygalaceae) is a reputable herb used in TKM for its memory enhancing effects. Radix Polygalae (RP), the root of *P. tenuifolia*, is used in more than half of the most famous Chinese prescriptions for increasing intelligence [40]. Polygalasaponins are the main constituents of this plant responsible for cognitive improvement. There is growing support for improved memory function in many animal models [41,42,43]. BT-11, an ethanolic extract of *Polygala* root, is effective against scopolamine-induced cognitive impairment in passive avoidance response and water maze tests [44], as well as improving stress-induced memory deficits in rats [45]. Most saponins in RP are derivatives of presenegenin. The hydrolysate of polygalasaponins ameliorates Aβ_(25−35)_ induced amnesia, probably via its strong antioxidant properties [46]. AChE activity is inhibited *in vitro* by an 80% ethanol extract of *P. tenuifolia* [41,47]. Tenuigenin, an active component extracted from a RP, improved learning and memory. Oral administration of tenuigenin markedly reduced AChE activity, and malondialdehyde levels, increased superoxide dismutase activity in the hippocampus, and improved performance in Y-maze tests. In electrophysiological tests of hippocampal brain slices, tenuigenin infusion substantially enhanced field excitatory postsynaptic potentials amplitude, both in basic synaptic transmission and after high frequency stimulation in the Schaffer to CA1 pathway [48]. Onjisaponin F, a constituent in *P. tenuifolia*, increased choline acetyltransferase mRNA levels in rat basal forebrain cells. Oral administration of tenuifolin extracted from RP evidently improved latency and the number of errors in aged mice. Moreover, tenuifolin at the same dose increases the relative levels of norepinephrine and dopamine in the hippocampus of aged mice, and decreases AChE activity in the cortex [43]. A root extract of *P. tenuifolia* also induces the proliferation of a stem cell population in the rat hippocampal CA1 region [49]. Another study investigated the cognitive enhancing effect of polygalasaponin XXXII (PGS32), a triterpenoid saponin isolated from the roots of *P. tenuifolia.* PGS32 showed cognition enhancing effects in a mice scopolamine model, possibly through improvements in synaptic transmission, activation of the mitogen-activated protein kinase cascade, and enhancement of brain-derived neurotrophic factor levels [50].

### 4.3. Ginkgo Biloba

*Ginkgo biloba* (Fam. Ginkgoaceae) is a living fossil tree, having undergone little evolutionary change for almost 270 million years. It is native to China and is also cultivated in other parts of World. It is mainly cultivated for its nuts and leaves. The most important constituents of standardized extracts of dried *G. biloba* leaves are flavone glycosides (quercetin, kaempferol, and isorhamnetin) and terpene lactones (ginkgolides and bilobalide) [51,52]. A standardized and popular commercial *G. biloba* extract called EGb 761 contains various biologically active constituents. This standardized extract is available in several commercial forms for treating or preventing claudication, cognitive decline, dementia, and cerebral insufficiency. This extract increases the rate of acetylcholine turnover and stimulates binding activity of ligands to muscarinic receptors in the hippocampus [53]. EGb 761 facilitated memory processes through effecting acquisition, performance, and retention, and also improved the retrieval of the learned response in an appetitive operant conditioning in mice [54]. According to earlier published reports *G. biloba* showed benefits in cognitive deficits and dementia [55,56]. It also has antioxidant and free radical scavenging activity in animals [57,58,59]. The multiple effects of *G. biloba* can be related to the various components present in the extract, which may act independently or synergistically.

Ginkgo has gone through various clinical trials; it was found to be effective in treating patients with AD and multi-infarct dementia [56], individuals between 55 and 86 years of age with no history of significant neurocognitive dysfunction [60], elderly patients with dementia (AD or VD) or age-associated memory impairment (AAMI) [61], in elderly individuals with normal cognition or those with MCI [62], and in middle-aged healthy volunteers age 45–56 years were [63]. Moreover, it was found to be safe and effective in a multi-center, double-blind, randomized, placebo-controlled, 24-week trial with 410 outpatients [64]. Recently, a study assesses the association between intake of EGb761 and cognitive function of elderly adults over a 20-year period and reported that cognitive decline was lower in the subjects using EGb761 [65]. However, some clinical studies have showed that *G. biloba* did not reduce the risk of progression to AD [66] and did not result in less cognitive decline in older adults with normal cognition or with mild cognitive impairment [67].

### 4.4. Acorus Gramineus

*Acorus gramineus* (Fam. Acoraceae) is listed in the Korean Pharmacopeia due to its several pharmacological activities, and has been used for hundreds of years for its beneficial effects on learning and memory, as well as in India and China for its anti-aging effects [68]. The main active compounds α-asarone and β-asarone, are the most promising candidates for eliciting cognitive improvement, and have been reported for their AChE inhibition effects *in vitro* [69]. Several studies have shown the cognitive enhancing effects of this plant. In our lab, α-asarone showed a reversal of scopolamine-induced amnesia, and inhibited AChE activity *in vivo* [70]*.* Chong–Myung–Tang (CMT) which consists of *A. gramineus*, *Polygala tenuifolia*, and *Poria cocos*, is one of the TKMs used for the therapy of learning and memory improvement. In a study, CMT showed cognitive improvement, via regulation of cholinergic marker enzyme activities and the antioxidant defense system [71]. Oral administration of the essential oil of *A. gramineus* improved the latency and number of errors in aged, dysmnesic rats and mice. Furthermore, there were increased levels of norepinephrine, dopamine and serotonin, and decreased levels of AChE activity in aged rats after treatment with essential oil of *A. gramineus* [72]. *A. gramineus* rhizoma (AGR) has been widely used as a herbal medicine against ischemia. AGR pretreatment produced a significant improvement in common tests used to assess memory like the Morris water maze and radial arm maze. Consistent with behavioral data, pretreatment with AGR significantly reduced ischemia-induced cell death in the hippocampal CA1 area. These results demonstrated that AGR have a protective effect against ischemia-induced neuronal loss, as well as learning and memory damage, and that AGR may be useful in the treatment of VD [73].

### 4.5. Green Tea

Green tea derived from *Camellia sinensis* (Fam. Theaceae) is a popular beverage in Asian countries like Korea, China, and Japan. The major polyphenolic constituents in green tea are epigallocatechin-3-gallate (EGCG), epicatechin, epigallocatechin and epicatechin-gallate. EGCG is the prominent and most active component of green tea catechins [74,75]. It acts as an antioxidant [76] and attenuates lipid peroxidation caused by various forms of free radicals in biological systems [77]. Tea leaves have also been reported for their cognition-enhancing ability [78] and for abating oxidative changes associated with aging [79]. Green tea may possess potent neuroprotection and amyloid precursor protein-processing activities, which may lead to cognitive enhancement. Green tea catechin potential in aging-impaired cognition and neurodegenerative diseases has been reviewed [80], and it might be used for disease modifying therapy in combination with other compounds. In one study, it was found that the chronic administration of tea polyphenols improved cognitive performance and inhibited AChE activity in scopolamine-induced amnesic mice [81]. In another study green tea extract administration was found to be effective in enhancing learning and memory in aged rats, and also demonstrated selectivity for inhibition of AChE [82]. In recent years, green tea has been explored in clinical trials relating to learning and memory-related disorders, indicating its potential for improving learning and memory [83]. In a randomized, double-blind, placebo-controlled study, a combination of green tea extract and L-theanine (LGNC-07) showed potential cognitive improvement in patients with MCI [84]. In some clinical studies, the consumption of green tea was assessed in older aged subjects, and it was concluded that tea consumers had better performance than non-consumers in several memory tests [85,86]. Cognitive impairment was associated with a lower frequency of tea consumption in men, but not women [87]. Total tea consumption (including both green tea and black/oolong tea) improves global cognition memory, executive function, and information processing speed performances [88].

### 4.6. Angelica

*Angelica gigas* (Fam. Umbelliferae) has been used in traditional Korean folk medicine, not only for the treatment of anemia, but also as a sedative, an anodyne, and as a tonic agent [89]. It has been recently reported that an extract of *A. gigas* and decursinol, a coumarin isolated from *A. gigas*, showed a high AchE inhibitory activity *in vitro* [90]. In a study it was found that the long-term oral administration of an ethanolic extract of *A. gigas* (EAG) or decursinol, showed beneficial effects on β-amyloid peptide Aβ_(1–42)_-induced memory impairment in mice. Pretreatment of mice with EAG (0.1%) and decursinol (0.001%, 0.002%, and 0.004%) for four weeks significantly blocked the Aβ_(1–42)_-induced impairment in passive avoidance performance. These findings suggest that EAG or decursinol may have a preventive effect against memory impairment related with AD [91]. *Angelica keiskei*, another variety of Angelica, has been used traditionally as a laxative, analeptic, diuretic and galactagogue in Korea. Recently, the regulatory effects of *A. keiskei* on memory impairment was investigated using Y-maze, passive avoidance, and the Morris water maze tasks. Furthermore, AChE activity assay was performed to investigate the cholinergic antagonistic effect of *A. keiskei* in the hippocampus. The effects of *A. keiskei* on the phosphorylation of CREB and on the expression of BDNF were evaluated by western blot and immunohistochemistry assays. The findings showed that *A. keiskei* significantly attenuated scopolamine-induced cognitive impairment in mice. An increase of AChE activity caused by scopolamine was significantly attenuated by *A. keiskei*. Additionally, *A. keiskei* significantly recovered the phosphorylation of CREB, and the expression of BDNF by scopolamine in the hippocampus. Taken together, these results suggests that *A. keiskei* might be a useful against learning and memory deficit caused by AD and aging [92]. In another study the effects of imperatorin [9-(3-methylbut-2-enyloxy)-7H-furo[3,2-g]chromen-7-one], a bioactive furanocoumarin isolated from the fruits of *Angelica officinalis*, was investigated for its effect on anxiety and memory-related behaviors of mice. Male Swiss mice were tested for anxiety and cognition, in the elevated plus maze test (EPM), using two different procedures. It was observed that, acute and repeated administration of imperatorin improved different stages of memory processes (both acquisition and consolidation) in a modified EPM test. These results suggest imperatorin to be an interesting therapeutic option in disorders with high anxiety levels and memory impairment [93].

## 5. Miscellaneous TKM Plants

*Poria cocos* (also known as *Wolfiporia extensa*; Fam. Polyporaceae) is a saprophytic fungus, which is used in Korea as well as other East Asian countries to treat age-related brain disorders, and to improve cognitive and memory function in old age [94]. It’s sclerotium, commonly known as “Hoelen”, contains several monosaccharides, triterpene derivatives, and 15 amino acids [95,96]. Poria has been reported to improve cerebral blood flow [97], promote hippocampal LTP *in vivo* [98], improve memory, and to inhibit AChE activity [99]. Poria has been used as an important constituent of Korean formulations like Chong-myung-tang, Palmul-chongmyung-tang, Guibi tang, BR003, and Kami-ondam-tang, for improving memory, as mentioned in Table 1.

*Cnidium officinale* (Fam. Apiaceae) has been used as a side dish and as a medicinal plant for a long time in Asia, especially in Korea and China. *C. officinale* has been used as a sedative and in the treatment of anemia. Sixty-eight volatile flavor constituents were detected in *C. officinale* essential oil, which contained hydrocarbons, aldehydes, alcohols, ketones, esters, oxides, acid and phthalides. *cis*-butylidenephthalide was the most abundant component followed by 3-butyl phthalide, *cis*-3-isobutylidenephthalide (10.1%) and terpinen-4-ol (8.5%) [100]. *C. officinale* has been shown to improve memory in scopolamine-injected mice [101]. Several formulations containing *C. officinale* have been showed to improve memory impairment in cerebral ischemia [102,103], Aβ_(25–35)_-injected mice [104] and Tg-APPswe/PS1dE9, transgenic mice of AD [105].

*Bupleurum falcatum* (Fam. Apiaceae) is a historical Korean plant which is used for the general improvement of cognition and memory function in old age, and a methanol extract of this plant has been previously shown to inhibit AChE by up to 24.7% [106]. *B. falcatum* has been used as a traditional medicine throughout the World, and is one of the major components of herbal treatments, and is used in the treatment of many psychosomatic disorders, including stress and mental illness [107]. The major pharmaceutical constituents of *B. falcatum*, including saikosaponin-a, saikosaponin-c, and saikosaponin-d aglycones, are located in the roots [108]. *B. falcatum* has been proven to be effective in enhancing cognitive function in recent pharmacological studies. Several studies have demonstrated the pharmacological effects of *B. falcatum* on the central nervous system through modulation of the HPA axis. *B. falcatum* has a protective effect against repeated immobilization stress induced neuronal and cognitive impairments, and they suggest that *B. falcatum* may be useful in the treatment of stress induced memory impairment [109].

*Pulsatilla koreana* (Korean pasque flower; Fam. Ranunculaceae) is one of the most important herbs in TKM and has been used for the treatment of amoebic dysentery and malaria. It has been reported to contain ranunculin, anemonin, protoanemonine, triterpenes and saponins. The ethanolic extract of *P. koreana* was found to have neuroprotective effects against Aβ_(1–42)_-induced neurotoxicity [110]. The oleanolic-glycoside saponins enriched fraction, designated as SK-PC-B70M, obtained from the *n*-BuOH fraction of *P. koreana*, improves scopolamine-induced impairments of memory consolidation and spatial working memory [111].

*Dioscorea* species (Fam. Dioscoreaceae) have traditionally been used for the treatment of memory-related diseases, such as AD and other neurodegenerative diseases. In one study, *in vivo* and *in vitro* tests were carried out to evaluate the cognitive enhancing effects of the CHCl_3_-soluble extract of *Dioscorea opposita* against scopolamine-induced amnesic mice and glutamate and H_2_O_2_-treated cortical neurons of rats. Results from this study suggest that *D. opposita* has neuroprotective effects on memory impairment related neurodegenerative diseases [112]. Yam (*Dioscorea pseudojaponica*) ameliorates cognition deficit and attenuates oxidative damage in senescent mice induced by D-galactose [113]. In a recent patent an extract of *D. opposita* having neuroprotective activity for preventing and treating brain disease was claimed. The extracts from *D. opposita* show potent neuronal cell protective activity by inhibiting neuronal cell death caused by neurotoxicity of glutamate and H_2_O_2_, and therefore it is suitable for treating and preventing brain diseases, whether consumed as a health food or further refined [114].

*Nelumbo nucifera* rhizome (NR) (Fam. Nelumbonaceae) is a popular herb in TKM. NR has been considered to be a demulcent, a diuretic, and a cholagogue and has been used to treat piles, dyspepsia, and diarrhea. In one study, the effect of NR extract on learning and memory function was investigated. The methanol extract of NR resulted in significant improvements of memory functions and neurogenesis in the DG. In the passive avoidance test, the retention time of NR-treated rats was significantly longer than that of the controls and there was increased cell proliferation and cell differentiation in DG as assessed using immunohistochemical analyses. These results propose that NR extract might improve learning and memory by increasing neurogenesis in the DG of the hippocampus [115].

## 6. Korean Herbal Formulations for Cognition

Synaptic modifications in the brain are responsible for memory, and alterations of synaptic modifications can cause memory impairment. To date, memory pharmacology which reverses deteriorated memory has not been well elucidated. Synaptic modification within memory traces have been linked to various neurotransmitters (acetylcholine, glutamate, γ-aminobutyric acid, serotonin, dopamine, histamine), neuromodulators (cannabinoid, opioid, corticosteroids, neuropeptides, BDNF), ions (calcium, potassium) and secondary messengers and enzymes (adenylcyclase, phospholipase, protein kinase) [116]. Numerous herbal plants used in TKM are combined in the form of multi-herbal formulas that are more effective than individual herbs alone. These herbal combinations are supposed to act synergistically to complement beneficial effects and to neutralize the toxic or adverse effects of individual constituent herbs. These formulas are based on traditional wisdom and experience, and have more recently been further validated by scientific reports which support their beneficial effect. Here, we summarized herbal formulations used in Korea for memory improvement in Table 1.

**Table 1:** Evidence-linked herbal formulations used in Korea for their memory-enhancing effects.

S.No.	Formulation	Traditional Plants	Experimental Evidence	Reference
**1.**	Chongmyeong-tang	*Acorus gramineus; Polygala tenuifolia;* *Poria cocos*	CMT may be useful for cognitive improvement via regulation of cholinergic marker enzyme activities and the antioxidant defense system	[71]
**2.**	Palmul-chongmyeong-tang (PMCMT)	*Panax ginseng; Atractylodis macrocephala;* *Poria cocos; Glycyrrhiza uralensis;* *Angelica gigas; Ligusticum chuanxiong; Rehmannia glutinosa; Paeonia albiflora; Acorus gramineus; Polygala tenuifolia*	Treatment with PMCMT reduced the loss of cholinergic immunoreactivity in the hippocampus induced by cerebral ischemia and improved memory. It might be useful for the treatment of vascular dementia	[117]
**3.**	Guibi-tang (GBT) (Quipi-tang or Kihi-To)	*Panax ginseng; Astragalus membranaceus; Atractylodes macrocephala; Poria cocos;* *Zizyphus jujube; Euphoria longan;* *Angelica sinensis; Polygala tenuifolia;* *Saussurea lappa; Glycyrrhiza uralensis*	GBT improves learning and memory but also increases the proliferation of cells in the DG of the hippocampus	[118]
**4.**	Kami-ondam-tang (KOT)	*Pinellia ternate; Phyllostachys nigra;* *Poncirus trifoliate; Poria cocos;* *Citrus unshiu; Glycyrrhiza uralensis;* *Polygala tenuifolia;* *Scrophularia buergeriana; Panax ginseng; Rehmannia glutinosa;* *Zizyphus jujuba var. spinosa;* *Zizyphus jujuba var. inermis;* *Zingiber officinale*	KOT administration significantly increased the expressions of phosphorylated Akt, phosphorylated CREB and BDNF in the hippocampal CA1 and dentate gyrus. In addition, KOT administration resulted in a significant increase in the number of DCX-immunopositive cells in the DG and improve memory	[119]
			KOT attenuates MK-801-induced PPI disruption, social interaction deficits, and cognitive impairments, possibly by regulating cortical Akt and ERK signaling	[120]
**5.**	Yeoldahanso-tang	*Pueraria lobata; Angelica tenuissima;* *Scutellaria baicalensis;* *Platycodon grandiflorum;* *Angelicae dahurica; Cimicifuga heracleifolia;* *Raphanus sativa; Polygala tenuifolia;* *Acorus gramineus; Dimocarpus longan*	Traditionally Yeoldahanso-tang been used to treat amnesia, hypochondria and dementia. It also showed neuroprotection in Parkinson’s disease	[121,122]
**6.**	Yukmijihwang-tang or Luweidihuang-wang (YMJ)	*Cornus officinalis; Rehmannia glutinosa; Poria cocos; Paeonia suffruticosa;* *Lycium chinense; Alisma orientalis*	YMJ prevents the deterioration of learning and memory ability in senescent accelerated mice and hydrocortisone-treated mice	[123]
			YMJ reverses scopolamine-induced and p-chloroamphetamine-induced amnesia in rats. YMJ cause significant reversal of ibotenic acid-induced deficit in learning and memory	[124,125]
			YMJ derivatives enhance cognitive ability in normal human subjects, accelerates the speed of information processing and enhances cognitive ability	[126]
**7.**	Paeng-jo-yeon-nyeon-baek-jain-hwan (PJBH)	*Dendrobium moniliforme; Thuja orientalis;* *Torilis japonica; Rubus coreanus;* *Cornus officinalis; Schizandra chinensis;* *Morinda officinalis; Asparagus cochinchinensis; Polygala tenuifolia;* *Phlomis umbrosa; Panax ginseng;* *Rehmannia glutinosa; Cinnamomum cassia; Acorus calamus; Alisma canaliculatum; Dioscorea japonica; Cistanche salsa*	PJBH has an extensive history in Korea, and is also used in TCM to activate brain function, promote memory and lengthen life span. PJBH have remarkable elevating effect on catalase and GSH-Px activities as well as cell survival	[127]
**8.**	Sipjeondaebo-tang	*Panax ginseng; Astragalus membranaceus; Atractylodes japonica; Paeonia albiflora;* *Angelica gigas; Cnidium officinale;* *Citrus unshiu; Glycyrrhiza uralensis; Polygonum multiflorum; Cinnamomum cassia*	SDT may participate in improvement of declined cerebral energy production and cholinergic neurotransmitter synthesis in senile dementia	[128]
**9.**	Gami-chunghyul-dan (GCD)	*Rheum palmatum; Scutellaria baicalensis;* *Gardenia jasminoides; Plantago asiatica;* *Cannabis sativa; Prunus humilis;* *Areca catechu*	GCD treatment rescues cognitive impairment induced by soluble oligomeric forms of amyloid beta (AβO) as well as protects against AβO-induced hippocampal cell loss. Moreover, GCD inhibits AβO-induced astrogliosis and microgliosis in the hippocampus, and protects against a decline in cholinergic synaptic density in the hippocampus of AβO-injected mice	[129]
**10.**	OSS (optimized-herbal formula from Sopung Sunkiwon)	*Bombyx mori; Plantago asiatica;* *Rheum palmatum; Poria cocos;* *Gardenia jasminoides; Cuscuta chinensis*	OSS has a protective effect against scopolamine-induced memory impairment in mice and increase synaptophysin and PSD-95, facilitating acetylcholine release and synaptic growth	[130]
**11.**	Kyung-ok-ko (KOK)	*Panax ginseng; Rehmannia glutinosa;* *Poria cocos; Lycium chinense;* *Aquillaria agallocha; Apis indica*	KOK significantly prevented scopolamine induced cognitive impairment and inhibited AChE dose dependently *in vitro* and *in vivo*	[131]
			KOK administration significantly attenuated ischemia-induced cognitive impairments in mice, as observed in the Y-maze and novel object recognition tasks	[132]
**12.**	LMK02-Jangwonhwan	*Panax ginseng; Acorus gramineus;* *Poria cocos; Angelica gigas;* *Ophiopogon japonicas;* *Scrophularia buergeriana; Thuja orientalis*	LMK02-Jangwonhwan partially suppressed oxidative stress accumulation, and prevented the down-regulation of phospho-CREB and calbindin typically seen in the hippocampus of AD-like brains	[133]
**13.**	LMK03-Jangwonhwan	*Poria cocos; Angelica gigas*	LMK03-Jangwonhwan has a potency to inhibit AD-like pathology at a detectable level, but LMK03 is not likely to retain the major ability of LMK02-Jangwonhwan to modify AD pathology in several AD related molecular parameters	[134]
**14.**	Cereboost	*Panax quinquefolius* standardized to 10.65% ginsenosides	Improvement in working memory performance, reaction time accuracy and calmness in healthy young volunteers	[135]
**15.**	LGNC-07	combination of green teaextract and l-Theanine	Significant improvement in selective attention, cognitive alertness, memory and verbal reading in mild cognitive impairment patients	[84]
**16.**	HT008-1	*Panax ginseng*; *Acanthopanax senticosus Angelica sinensis; Scutellaria baicalensis*	HT008-1 showed beneficial effect for cognitive improvement in healthy volunteers in controlled double-blind, placebo-controlled, randomized clinical trial	[136]
**17.**	BR003	*Acanthopanax senticosus;* *Schisandra chinensis; Ginkgo biloba; Panax ginseng; Astragalus membranaceus; Atractylodes macrocephala; Poria cocos;* *Ziziphus jujube; Euphoria Longan;* *Angelica sinensis; Polygala tenuifolia;* *Glycyrrhiza uralensis*	BR003 improves learning and memory and also increases cell proliferation in the DG of the rat hippocampus	[137]
**18.**	ESP-102 (Jamdanggwi)	*Angelica gigas; Saururus chinensis;* *Schizandra chinensis*	Acute oral treatment of mice with ESP-102 significantly reduced scopolamine-induced memory deficits in the passive avoidance performance test	[138]
**19.**	Cistanches Herba (CHE)	*Cistanche deserticola*	CHE improved memory through the stimulation of NGF secretion, increased neuronal cell differentiation, neurite length, and synapse formation in the mouse hippocampus. CHE is useful for improving memory function via its action in upregulating nerve growth factor	[139]
**20.**	SK-PC-B70M	*Pulsatilla koreana*	SK-PC-B70M has effects on reversing impairments of memory consolidation and working memory impairments induced by scopolamine	[111]
			SK-PC-B70M has a beneficial effect on the Tg2576 murine model of AD through reducing soluble and insoluble forms of Aβ_(1mode_ and anti-oxidant activity	[140]
**21.**	BT-11	An ethanolic extract from the *Polygala tenuifolia* root	BT-11 improved scopolamine and stress induced amnesia in rats and has memory-enhancing effects in healthy adults	[44,45,141]
**22.**	EGB-761 ^*^	Standardized extract of *Ginkgo biloba*	Significantly showed improvement in the speed of processing abilities in aged subjects with no history of significant neurocognitive dysfunction	[60]
			Effective in Alzheimer’s treatment and multi-infarct dementia in patients with AD and multi-infarct dementia	[56]
**23.**	GK501 ^*^	Standardized extract of *Ginkgo biloba*	Improvement in the speed of attention and tasks associated with episodic memory performance	[142]
**24.**	G115 ^*^	Standardized extract of *Panax ginseng*	Improvement in speed of attention and tasks associated with episodic memory performance in healthy middle aged individuals	[143]
**25.**	Perilla diet	*Perilla frutescens*	Perilla diet supplementation promotes neuronal signaling and alters synaptic plasticity for improved learning and memory	[144]
**26.**	INM 176 (K-1107)	Ethanolic extract of *Angelica gigas*	INM 176 showed benefits in placebo-controlled clinical trial in the old aged subjects with memory impairment	[145]
			INM-176 inhibited AChE activity in the hippocampal tissue *in vitro* and *ex vivo*. Single or subchronic administration of INM-176 attenuated scopolamine or Aβ_(1–42)_ induced cognitive dysfunction	[146]

* Standardized extracts of Asian traditional medicine developed in Europe.

## 7. Conclusions

Traditional medicine constitutes the backbone of medical practices in many Asian countries, most notably Korea and China. In pathologies involving cognitive impairment (AD, VD, MCI) the homeostatic balance essential for normal functioning of the cellular cascade is disturbed. Most memory deficit problems are associated with disruption of the cholinergic pathway, however several other factors also play roles which ultimately lead to a decline in synaptic plasticity as well as cognitive decline. Numerous herbal plants which are used in TKM are combined in the form of multi-herbal formulas that are believed to be more effective than the individual herbs alone. These herbal combinations are supposed to act synergistically. These formulas are based on traditional wisdom and experience, and have recently been the subject of scientific reports which support their beneficial effects. Moreover, with continuing scientific advances, these herbal formulations are receiving more and more attention across the World. Some traditional beliefs about the use of herbal medicines for improving memory have been supported by both *in vitro* and *in vivo* studies, as well as clinical studies. Future strategies should be focused to coordinate Western medicine with Traditional medicine. As evident with Korean statistics, the number of elderly will increase in coming decades, along with increases in a specific suite of health problems. TKMs, used either alone or in combination with conventional medicine, improve memory or have promised efficacy in clinical trials. We cautiously propose that the safety and efficacy of TKMs has been proven over thousands of years. In the near future TKM might deliver more products similar to ginseng and polygala for learning and memory improvement.
